# Effects of drying-rewetting cycles on the fluxes of soil greenhouse gases

**DOI:** 10.1016/j.heliyon.2023.e12984

**Published:** 2023-01-14

**Authors:** Xia Jin, Fuzhong Wu, Qiqian Wu, Petr Heděnec, Yan Peng, Zheng Wang, Kai Yue

**Affiliations:** a Key Laboratory for Humid Subtropical Eco-Geographical Processes of the Ministry of Education, School of Geographical Sciences, Fujian Normal University, Fuzhou 350007, China; b State Key Laboratory of Subtropical Silviculture, Zhejiang A & F University, Lin'an 311300, China; c Institute of Tropical Biodiversity and Sustainable Development, University Malaysia Terengganu, Kuala Nerus, 21030, Terengganu, Malaysia; d College of Forestry, Hebei Agricultural University, Baoding 071000, China; e Fujian Sanming Forest Ecosystem National Observation and Research Station, Sanming 365002, China

**Keywords:** CO_2_ emission, CH_4_ flux, N_2_O flux, Experimental setting, Soil property, Meta-analysis

## Abstract

Irregular precipitation caused by climate changes has resulted in frequent events of soil drying-rewetting cycles (DWC), which can strongly affect soil carbon (C) and nitrogen (N) cycling, including the fluxes of greenhouse gases (GHGs). The response of soil carbon dioxide (CO_2_), methane (CH_4_), and nitrous oxide (N_2_O) fluxes to DWC events may differ among different ecosystem types and vary with experimental settings and soil properties, but these processes were not quantitatively assessed. Here, we evaluated the responses of soil GHG fluxes to DWC, compared with consistent moisture, as well as the associated driving factors with 424 paired observations collected from 47 publications of lab incubation experiments. Results showed that: (1) DWC significantly decreased soil CO_2_ emissions by an average of 9.7%, but did not affect the emissions and uptakes of soil CH_4_ and N_2_O; (2) DWC effects on soil GHG emissions varied significantly among different ecosystem types, with CO_2_ emissions significantly decreased by 6.8 and 16.3% in croplands and grasslands soils, respectively, and CH_4_ and N_2_O emissions significantly decreased and increased in wetlands and forests soils, respectively; (3) the effects of DWC on CO_2_ emissions were also positively regulated by organic C and N concentrations, pH, clay concentration, and soil depth, but negatively by C:N ratio and silt concentration, while DWC effects on N_2_O emissions were negatively controlled by C:N ratio, silt concentration, and soil depth. Overall, our results showed that CO_2_ emissions were significantly decreased by DWC, while the fluxes of CH_4_ and N_2_O were not affected, indicating an overall decrease of GHGs in response to DWC. Our results will be useful for a better understanding of global GHG emissions under future climate change scenario.

## 1 Introduction

Global climate change has leaded to severe alterations in precipitation regimes that significantly increase in some areas but decrease in the others [1], resulting in drying-rewetting cycles (DWC) in soils that affect soil carbon (C) and nitrogen (N) cycling substantially [2,3]. Drought caused by reduced precipitation always leads to decrease in microbial activity and soil respiration [4], and the following rewetting would stimulate microbial biomass and activity, bursting a large proportion of gaseous C and N from soils, such as carbon dioxide (CO_2_), nitrous oxide (N_2_O), and methane (CH_4_). This process is defined as the “Birch Effect” [5], which can be interpreted by two main driving mechanisms. The physical mechanism is that soil microorganisms rapidly consume the exposing soil organic matter (SOM) of previously physically protected after disruption of soil aggregates [6,7]. The physiological mechanism means that compatible solutes produced by microorganism and accumulated in cells in order to maintain the balance of osmotic pressure under drought pressure, and it would be disposed rapidly by itself under rewetting to prevent membrane rupture [8,9]. Rewetting of dry soils provides an ideal condition in water films connectivity for microorganisms to access substrates for living, which can stimulate a large of soil GHG pulses [10]. Emissions of these greenhouse gases (GHGs) into the atmosphere will further influence global climate change.

Studies reported that the response of soil CO_2_ emissions to one or multiple times of DWC increased compared to that in soil with constant moisture [11,12]. However, it is worth noting that the high CO_2_ emissions after rewetting is maintained only a few days if the moisture days are too long, resulted from the depletion of substrate [13,14]. Evidence also showed that CO_2_ emissions significantly decreased under DWC stress because of the unequal balance of CO_2_ emissions between reduction during drought phase and pulses during rewetting phase [16]. Drought can explicitly inhibit the progress of nitrification and denitrification mediated by microbial, resulting in the reduction of N_2_O emission [15], while rewetting can stimulate the mineralization of SOM [16] as well as denitrification [17], resulting in the increase of N_2_O emissions. In addition, as affected by DWC, there will be a change in soil water table that influences process in decomposition and the capacity of available electron acceptor used for heterotrophic respiration [18,19], and aerobic conditions caused by drought resulted in inhibition of CH_4_ emissions, whereas rewetting period provides a anaerobic condition for CH_4_ production [20]. However, we still lack a clear perspective on the patterns of the effects of DWC on soil GHG fluxes.

The effects of DWC on soil GHG fluxes may vary among different types of ecosystems. For example, a study found that increases in CO_2_ emissions stimulated by DWC were higher in forests than in desserts soils, which were attributed to different concentrations of SOM [12]. Also, the effect of DWC in the same ecosystem type can have divergent effects on different GHGs. For instance, CO_2_ emissions significantly increased but N_2_O emissions decreased after two times of DWC in grassland soils, likely because a constant moisture of soils can positively affected N_2_O emissions and repeated DWC may affect the activities of microorganisms [21], while DWC was observed to significantly decrease CH_4_ emissions but do not affect CO_2_ emissions in wetlands soils [22].

The effects of DWC on soil GHG fluxes can be also modulated by soil properties such as the concentrations of soil organic C (SOC) and N, soil texture, pH, and C:N ratio, because these variables are directly or indirectly related to the production or uptake of soil GHGs. For instance, N_2_O emissions in clay loam soils were found to be higher than silt loam soils under DWC stress, but CO_2_ emissions were higher in silt loam soils compared with clay loam soils [23]. Also, the response of soil inorganic N to experimental drought in sandy loam soil with higher SOC concentration were greater than the soil with low SOC concentration [24]. Also, experimental setting such as the numbers of DWC, total incubation day, duration of drying and rewetting, initial water holding capacity (WHC), and incubation temperatures can be also important moderator variables for the effects of DWC on GHG fluxes [9,16]. For example, the CO_2_ pulse following rewetting usually reduced or even faded with increasing numbers of DWC [25]. However, till now, there is not a clear perspective on the impacts of moderator variables on the effects of DWC on soil GHG fluxes.

Here, to better understand how DWC may affect the fluxes of soil GHGs, we quantitatively evaluated the effects of DWC on soil CO_2_ emissions, N_2_O emissions and uptakes, and CH_4_ emissions and uptakes with 424 paired observations collected from 47 publications. The objectives of this study were to (1) quantify the effects of DWC on the fluxes of soil CO_2_, N_2_O, and CH_4_ as a whole and within different types of ecosystems; and (2) evaluate the impacts of multiple moderator variables on the effects of DWC on soil GHG fluxes. We hypothesized that (1) DWC will increase the emissions of CO_2_, N_2_O, and CH_4_, but decrease the uptakes of N_2_O and CH_4_ because of frequent changes in soil moisture; and (2) the effects of DWC would be regulated by several moderator variables such as ecosystem types, soil properties, and experimental setting.

## 2 Methods and materials

### 2.1 Data

We searched peer-reviewed articles and academic theses published in English and Chinese on *Web of Science* and *China National Knowledge Infra*structure (CNKI) on July 10, 2022 with the search terms of (“carbon dioxide” OR CO_2_ OR methane OR CH_4_ OR “nitrous oxide” OR N_2_O OR “greenhouse gas*”) AND (rewet* OR drying-rewetting OR dry-rewet). To be included in our study, primary studies must meet the following criteria: (i) at least one of the assessed GHGs variables in response to DWC was reported; (ii) experiments should include both a constant moisture control and a DWC treatment, and were established within the same experimental condition except for soil moisture; (iii) experiments must contain at least three replication; and (iv) the means, sample sizes, standard deviations (SDs) or standard errors (SEs) of the assessed GHGs variables were directly reported or could be calculated. To evaluate the effects of moderator variables such as experimental setting and soil properties on the responses of GHGs to DWC, we also extracted data of soil incubation day, number of DWC, the ratio of drying days over total incubation days in a DWC (D:T ratio), the ratio of rewetting days over total incubation days in a DWC (R:T ratio), incubation temperature, soil moisture expressed as percent WHC in the control group, soil sampling depth, SOC, N, C:N ratio, pH, and the concentrations of clay, silt, and sand, where available. Because all the data included in our study were collected from lab incubation experiments, factors such as climate, latitude, and elevation were not assessed, but the effects of ecosystem type (cropland, desert, forest, grassland, rice paddy, and wetland) from which soil samples were collected was evaluated.

**Fig. 1:**
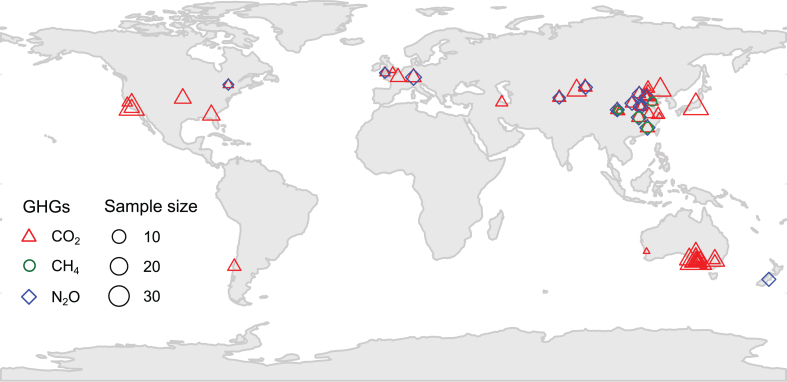
Global map showing the distribution of study sites from the 47 articles in the meta-analysis. The number of observations for each site is represented by symbol size, and the greenhouse gas type is indicated by color. (For interpretation of the references to color in this figure legend, the reader is referred to the Web version of this article.)

After extraction, a total of 424 paired observations (347 for CO_2_, 16 for CH_4_, and 61 for N_2_O) from 47 publications that were all carried out as lab incubation study satisfied the criteria and were then included in our analyses (Fig. 1, Appendix 1). With meta-analytic residuals following previous studies [26,27], we tested the potential publication bias of our database. Results from Egger's regression, funnel plot, and trim-and-fill tests suggested non or limited publication bias (Table S1, Fig. S1), indicating that the primary studies included in our database are a representative sample of the available studies in the literature.

### 2.2 Statistical analysis

To quantify the effects of DWC on the fluxes of soil GHGs, we used the natural log response ratio (lnRR) as the standard effect size [28], and the individual lnRR for each paired observation as calculated using Eq. (1):

(1) $$lnRR=\ln\left(\frac{{\bar{X}}_{t}}{{\bar{X}}_{c}}\right)$$

where ${\bar{X}}_{t}$ and ${\bar{X}}_{c}$ are the means of a response variable in the treatment (DWC) and control (constant moisture) groups, respectively. The variance (*v*) associated with each lnRR was calculated by Eq. (2) [29]:

(2) $$v=\frac{s_{t}^{2}}{n_{t}{\bar{X}}_{t}^{2}}+\frac{s_{c}^{2}}{n_{c}{\bar{X}}_{c}^{2}}$$

where *s*_*t*_ and *s*_*c*_ are the SDs, and *n*_*t*_ and *n*_*c*_ are the sample sizes of a response variable in the treatment and control groups, respectively. The weight for each lnRR (*w*) was then calculated as the reciprocal of its variance (1/*v*).

To calculate the overall weighted effect sizes (lnRR_++_) of DWC on the fluxes of GHGs, we ran an intercept-only linear mixed-effects model for each GHG variable using the *lme4* package in R [30]. In each linear fixed-effects model, lnRR of GHG was fitted as the response variable, and the identity of primary studies from which data were extracted was fitted as a random effect factor to account for potential dependence among observations collected from a single primary study [31]. Then, the effects of moderator variables (experimental setting and soil properties) on the responses of GHGs to DWC were assessed using linear mixed-effects models by fitting each variable as a fixed effect factor. In addition, to aid the interpretation of results, lnRR_++_ and the associated 95% confidence intervals (CIs) were back-transformed to percentage changes using the equation $\left(e^{lnRR_{++}}-1\right)\times 100$. The effect of DWC is significant when the 95% CI does not overlap with zero. All the statistical analyses were performed in R version 4.1.1 [32].

## 3 Results

### 3.1 Effects of DWC on the fluxes of GHGs

**Fig. 2:**
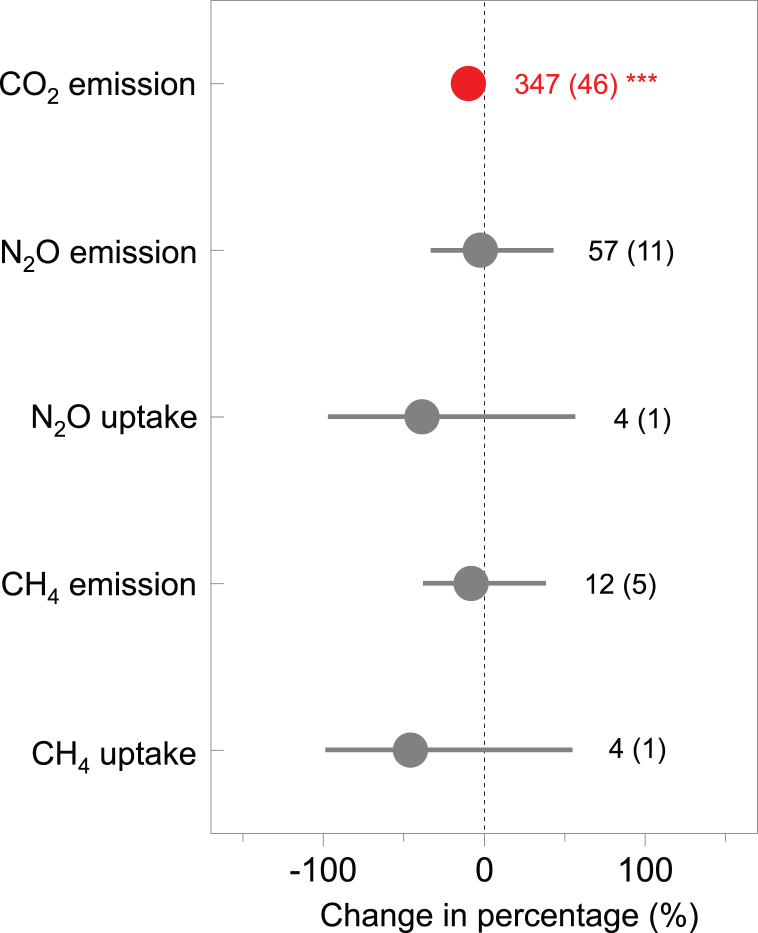
Mean effects of drying-rewetting cycles (DWC) on the fluxes of CO_2_, N_2_O, and CH_4_ overall. Values are mean ± 95% confidence intervals (CIs). The impact of DWC is significant when the 95% bootstrap CI does not overlap with zero. Numbers of paired observations are shown in brackets, and red indicates significantly negative effects. ***p < 0.001. (For interpretation of the references to color in this figure legend, the reader is referred to the Web version of this article.)

**Fig. 3:**
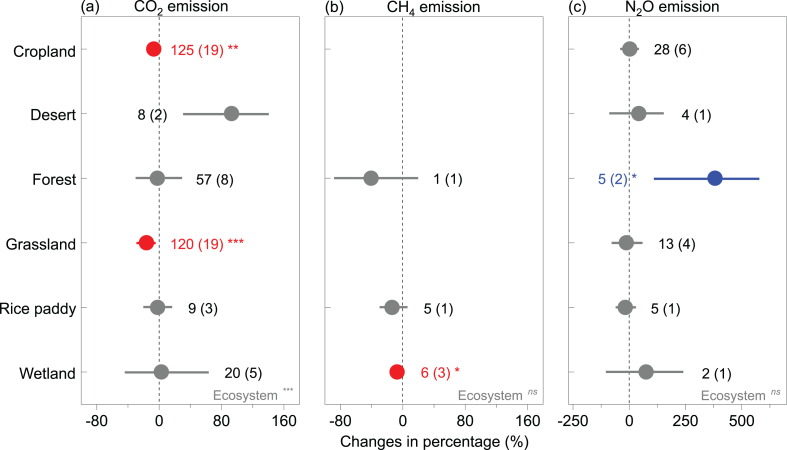
Effects of drying-rewetting cycles on the emissions of CO_2_, CH_4_, and N_2_O within different types of ecosystems. Values are mean ± 95% confidence intervals (CIs). The impact of DWC is significant when the 95% bootstrap CI does not overlap with zero. Numbers of paired observations are shown in brackets, and blue and red indicate significantly positive and negative effects, respectively. *p < 0.05, **p < 0.01, ***p < 0.001. (For interpretation of the references to color in this figure legend, the reader is referred to the Web version of this article.)

Averaged across all the observations, DWC significantly decreased soil CO_2_ emissions by 9.7%, but did not affect the emissions or uptakes of CH_4_ and N_2_O (Fig. 2). When assessed among different ecosystem types, the effects of DWC on CO_2_ emissions were only significant in croplands and grasslands soils, with average decreases of 6.8% and 16.3%, respectively, while CH_4_ emissions were significantly decreased by 7.4% under the stress of DWC in wetlands soils (Fig. 3). Also, the effects of DWC on N_2_O emissions were only significant in forests soils, with an average increase of 381.4%.

### 3.2 Impacts of moderator variables on the effects of DWC

**Fig. 4:**
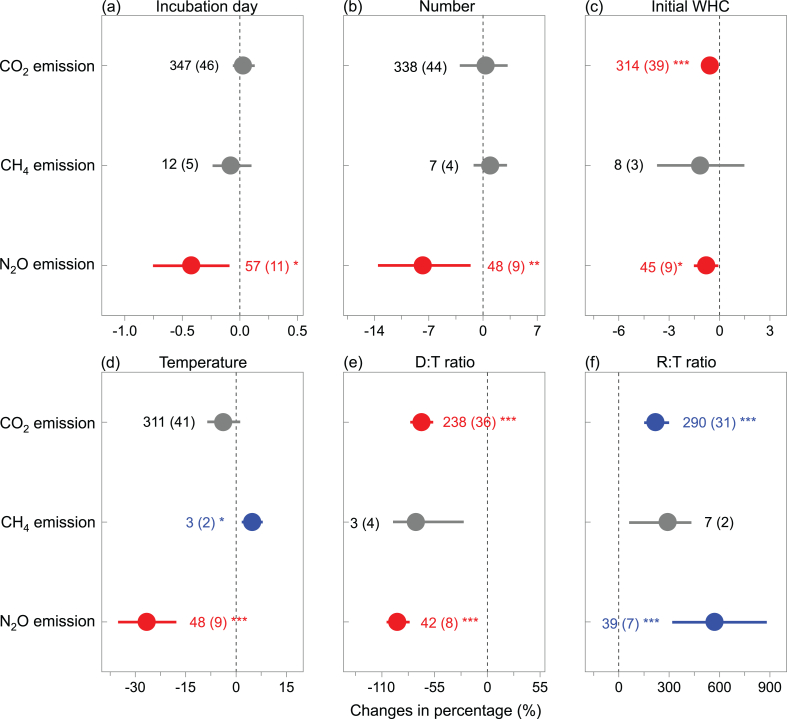
Effects of incubation day, number of drying-rewetting cycles, and the initial water holding capacity (WHC), incubation temperature, the ratio of drying days over total incubation days in a DWC (D:T ratio), and the ratio of rewetting days over total incubation days in a DWC (R:T ratio) on the responses of CO_2_, CH_4_, and N_2_O emissions to drying-rewetting cycles. Values are mean ± 95% confidence intervals (CIs). The impact of DWC is significant when the 95% bootstrap CI does not overlap with zero. Numbers of paired observations are shown in brackets, blue and red indicate significantly positive and negative effects, respectively. *p < 0.05, **p < 0.01, ***p < 0.001. (For interpretation of the references to color in this figure legend, the reader is referred to the Web version of this article.)

Both incubation day and the number of DWC showed significantly positive effects on the response of N_2_O emissions to DWC (Fig. 4a and b). The initial soil WHC (i.e., soil WHC in the control group) showed negative effects on the response of CO_2_ and N_2_O emissions to DWC, but had no effect on that of CH_4_ emissions (Fig. 4c). Also, incubation temperature showed positive effects and negative effects on the response of CH_4_ emissions and N_2_O emissions to DWC, respectively (Fig. 4e), while D:T ratio and R:T ratio was negatively and positively related to the response of CO_2_ and N_2_O emissions to DWC, respectively (Fig. 4f and d). As to soil physicochemical properties, SOC, N concentration, pH, clay concentration, and soil depth were positively related to the effect size of DWC on CO_2_ emissions, but C:N ratio as well as silt concentration had negative effects (Table 1). In addition, soil C:N ratio, silt concentration, and soil depth were negatively related to the effect size of DWC on N_2_O emissions (Table 1).

**Table 1:** Effects of moderator variables on the responses of CO_2_, CH_4_, and N_2_O emissions to drying-rewetting cycles. Estimates and the number of observations were reported. Bold indicates significant effects.

Variable	lnRR of CO_2_ emission		lnRR of CH_4_ emission		lnRR of N_2_O emission	
	Estimate	n	Estimate	n	Estimate	n
Soil organic carbon (%)	0.016***	258	0.226	10	0.032	55
Nitrogen (%)	3.526***	258	−0.923	10	0.420	47
C:N ratio	−0.025***	146	−0.204	4	−0.048*	29
pH	0.193***	281	0.039	10	−0.007	55
Clay (%)	0.023***	244			−0.015	47
Silt (%)	−0.013***	244			−0.033*	47
Sand (%)	0.003	244			0.008	47
Soil depth (cm)	0.011**	280			−0.026*	36

## 4 Discussion

In contrast to our hypothesis, results showed that DWC significantly decreased CO_2_ emissions at the global scale, indicating that the CO_2_ pulse in rewetting phase cannot fully compensate the deficiency of CO_2_ emissions during the drought periods as a whole [23,33]. The pulse of CO_2_ during rewetting phase will reduce and even vanish with increasing DWC, probably due to the exhaust of available substrates, disruption of soil aggregates, as well as the destruction of composition and function of microbes [9]. Previous research suggested that the cumulative CO_2_ in the second cycle will be influenced by substrates in the first cycle [34], and the decreasing pulses of CO_2_ may be related to the death of proportion of microbial communities [16]. Our results, in contrast to the hypothesis, also suggested the non-significant changes in N_2_O fluxes under DWC stress. It is generally believed that the responses of N_2_O to DWC during the drying and rewetting phases usually result in diverse outcomes and can offset each other [35]. The reduction of transformation process of inorganic N caused by drought [15] can be impaired by increase in initial nitrification caused by more aerobic conditions with drought, and then rewetting can strongly stimulate the microbial activity and promote the mineralization of SOM [16].

The response of GHG fluxes to DWC can significantly vary among different ecosystem types. The responses of CO_2_ emissions to DWC significantly decreased in croplands and grasslands soils, likely due to the high rates of litter decomposition in grasslands and croplands [36] that reduce the capacity of microbes to resist repeated drying and wetting cycles [37,38]. Wetlands have relatively high SOC concentration, and its anaerobic environment is favorable for methanogens [39], resulting in large emissions of CH_4_. While the decline of water table in drying phase during DWC treatment caused a reduction of CH_4_ emissions [22]. The effects of DWC on N_2_O emissions were only significant in forests soils, which may be attributed to the thicker organic layer that has higher SOM concentrations compared with other ecosystem types, which plays an important role in resisting the repeated stress of changes in water moisture because of the hydrophobic property of SOM [16,38]. However, it is noteworthy that the observations for CH_4_ and N_2_O fluxes are relatively small, and further data are needed for more robust results. While the non-significant effects of DWC on N_2_O emissions in wetland may be contributed to the small sample size that limited the statistical power [40].

Our results suggested that the number of DWC had no impacts on CO_2_ emissions, because evidence showed that microbial communities experiencing repeated DWC were found to be more resistant to such stresses compared to constant moisture [41]. Also, previous research suggested that microbial activities during drying phase of the later DWC treatment may be also influenced by the availability of substrates, especially a longer rewetting period in former cycle [34]. Our results showed that the response of N_2_O emissions to DWC decreased with increasing numbers of DWC, likely due to that inorganic N is more efficiently to be used by microorganisms, which caused the decrease of cumulative inorganic N during the DWC treatment after the second cycle [13]. The effects of DWC on CO_2_ and N_2_O emissions were negatively related to soil WHC, probably due to the substrate limitation, which was supported by previous finding that substrate limitation was a key factor when osmotic potentials exceed −0.6 MPa, through studying the impact of soil moisture on the activity of nitrifying bacteria [42]. Similarly, soil cumulative respiration after DWC in upper constant moisture treatment (UC) are significant decreased because of approaching the optimum level [9].

The effects of DWC on CO_2_ and N_2_O emissions was negatively related to the D:T ratio, likely because of the disproportion of decreases during the dry phase and fluxes during the rewetting phase. The diminution of soil CO_2_ during the long drying duration exceed the CO_2_ pulses during rewetting duration [9]. Although some studies have indicated that N_2_O hot moments after rewetting are more intense when previous conditions are drier, the response of N_2_O hot moments will decrease if drought duration is too long, likely because that microorganisms enter a deeper state of dormancy [43]. At the same time, the effects of DWC on CO_2_ and N_2_O emissions was also positively related to the R:T ratio, which was a part of the cascading response, including the start of increasing DOC concentrations that followed by increased microbial biomass [14]. Interestingly, we also found that the higher incubation temperatures, the lower N_2_O emissions but higher CH_4_ emissions under DWC stress. The underlying mechanisms may be that higher incubation temperatures can lead to decrease in soil moisture but increase in oxygen concentration that are closely related to microbial activities, which impaired the denitrification process [44] to a certain extent and thus reduced N_2_O emissions. The larger CH_4_ emissions under higher soil moisture may be contributed to the stimulated microbial activities [20,22] rather than decreased soil moisture.

In terms of soil properties, we found that the effects of DWC on CO_2_ emissions was positively related to SOC, which can be explained by that the characteristics of SOM such as hydrophobicity, high aggregate stability, and high substrate availability can contribute to a greater capacity to resist multiple DWC [12]. Also, the effects of DWC on CO_2_ emissions was positively correlated with soil N but negatively with C:N ratio, likely because low C:N ratio and high soil N provides a serviceable N concentration facilitate microbial communities and SOM decomposition [45,46]. Increase in pH at a certain level is advantageous to weaken the linkage between SOM and mineral surfaces [47], and available for microbes to acquire previously protected SOM. In addition, our results showed that clay and silt concentrations have significantly positive and negative correlations with DWC effects on CO_2_ emissions, respectively, likely due to the greater potential of releasing substrates after physical disruption in clay soil than coarse-texture soils [48]. Although evidence has showed that DWC can result in large N_2_O emissions in finer texture soils with crucial conditions, such as sufficient available C, and a higher water-filled pore space [23], our results suggested that the response of N_2_O emissions was not affected by clay-texture soils, likely because of the limitation of experimental conditions in our studies. Our analysis also found that the effects of DWC on CO_2_ emissions was positively related to soil sampling depth, probably because that the main occupation of starvation-tolerant microbial communities are in deep soils [49,50]. In contrast, the response of N_2_O emissions to DWC was negatively related to soil sampling depth, likely because soil water content usually decrease with soil depth, which limited the process of nitrification and denitrification [15].

## 5 Conclusions

Using meta-analysis method, we found that repeated DWC significantly decreased soil CO_2_ emissions by an average of 9.7%, indicating that the CO_2_ pulses during rewetting phase cannot fully compensated the loss of CO_2_ during the drying period. The effects of DWC on CO_2_ emissions were significantly affected by ecosystem type, with 16.3 and 6.8% increases in CO_2_ emissions in soils of grasslands and croplands, respectively, while CH_4_ and N_2_O emissions were significantly decreased and increased in wetland and forest soils, respectively. The effects of DWC on CO_2_ and N_2_O emissions was negatively correlated with D:T ratio and positively corelated with R:T ratio, respectively, while the number of DWC significantly reduced the effects size of DWC on N_2_O emissions. In addition, soil silt concentration showed negative impacts on the response of CO_2_ and N_2_O emissions to DWC. Overall, our study clearly showed how DWC may affect the fluxes of soil GHGs across different ecosystem types and gradients of soil properties, which help us to better understand the responses of soil GHGs under future climate change scenarios.

## Author contribution statement

Kai Yue; Xia Jin: Conceived and designed the study; Analyzed and interpreted the data; Wrote the paper.

Xia Jin; Qiqian Wu; Petr Heděnec; Yan Peng; Zheng Wang; Fuzhong Wu: Analyzed and interpreted the data; Wrote the paper.

Fuzhong Wu; Kai Yue: Contributed to materials and analysis tools; Wrote the paper.

## Funding statement

K.Y. was funded by the 10.13039/501100001809National Natural Science Foundation of China (32271633) and the State Key Laboratory of Subtropical Silviculture (SKLSS-KF2022-02). Y.P. received funds from the 10.13039/501100001809National Natural Science Foundation of China (32201342) and 10.13039/501100003392Natural Science Foundation of Fujian Province (2022J01642), F.W. was founded by the 10.13039/501100001809National Natural Science Foundation of China (32171641), and Z.W. was founded by the 10.13039/501100003787Natural Science Foundation of Hebei Province (C2019204351).

## Data availability statement

Data associated with this study has been deposited at https://doi.org/10.6084/m9.figshare.21802728.v1

## Declaration of interest's statement

The authors declare that they have no known competing financial interests or personal relationships that could have appeared to influence the work reported in this paper.
