# Supplementary material for: Effects of drying-rewetting cycles on the fluxes of soil greenhouse gases
Source: Heliyon. 2023 Jan 14;9(1):e12984. doi: 10.1016/j.heliyon.2023.e12984 (PMC9871208; doi:10.1016/j.heliyon.2023.e12984)
Supplement: Multimedia component 1 [file mmc1.pdf]

Supporting Information

Table S1 Results of publication bias analysis using Egger's regression tests on the meta-analytic residuals and trim-and-fill tests from the multi-level meta-analytical model.

Egger's regression		Trim-and-fill test			
t-value	*p*-value	*p*-value for H_0	Estimate	Lower 95% CI	Upper 95% CI
–1.345	0.179	0.250	0.011	–0.022	0.044

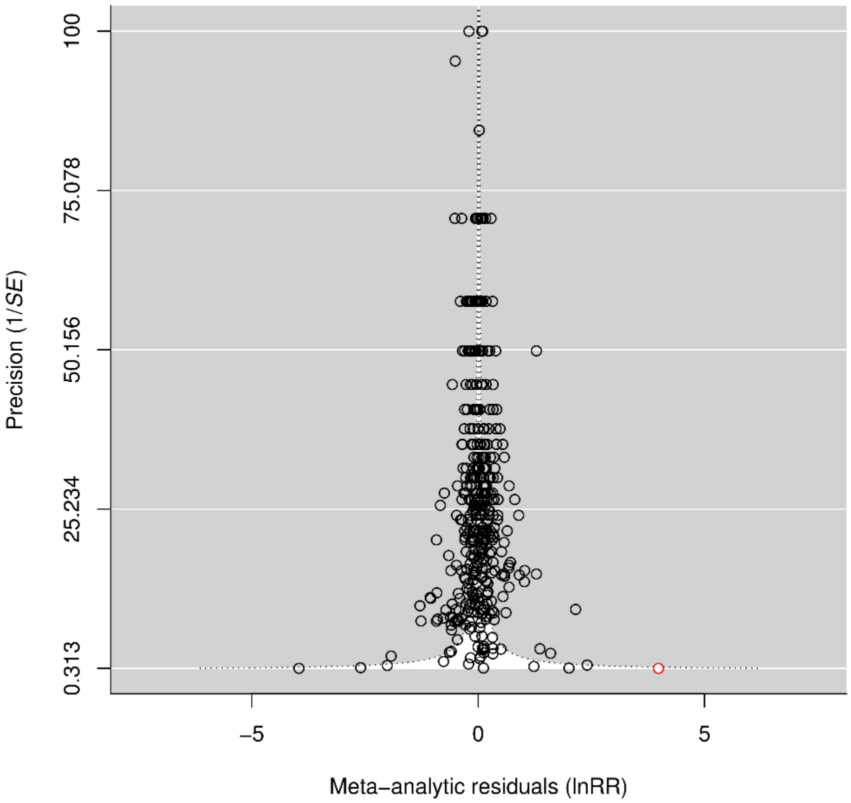

Fig. S1 Funnel plot displaying the residuals from the mixed-effect model plotted against the inverse standard error (precision) of invertebrate effects. Tests for publication bias were applied to the residuals. The solid vertical line indicates the mean of residuals, and white areas depict the space in which 95% of effects are expected to fall in the absence of heterogeneity or publication bias. Missing points were identified by a trim-and-fill test on the funnel plot.

Appendix 1 A list of the primary studies from which data were extracted.

1. Bagheri-Novair, S., Mirseyed Hosseini, H., Etesami, H., Razavipour, T., Asgari Lajayer, B., & Astatkie, T. (2020). Short-term soil drying–rewetting effects on respiration rate and microbial biomass carbon and phosphorus in a 60-year paddy soil. 3 Biotech, 10(11), 1-11.
2. Bao, Z. Z., Hou, Y. Y., & Zhu, X. P. (2018). Effect of alternating wetting and drying and simulated nitrogen deposition on soil CO_2 emission in alpine wetlands of Bayinbulak. Journal of Agro-Environment Science, 37(3), 598-604. (in Chinese with English abstract)
3. Beare, M. H., Gregorich, E. G., St-Georges, P. (2009). Compaction effects on CO_2 and N_2O production during drying and rewetting of soil. Soil biology and biochemistry, 41(3), 611-621.
4. Butterly, C. R., Bünemann, E. K., McNeill, A. M., Baldock, J. A., & Marschner, P. (2009). Carbon pulses but not phosphorus pulses are related to decreases in microbial biomass during repeated drying and rewetting of soils. Soil Biology and Biochemistry, 41(7), 1406-1416.
5. Butterly, C. R., Marschner, P., McNeill, A. M., & Baldock, J. A. (2010). Rewetting CO_2 pulses in Australian agricultural soils and the influence of soil properties. Biology and Fertility of Soils, 46(7), 739-753.
6. Butterly, C. R., McNeill, A. M., Baldock, J. A., & Marschner, P. (2011). Rapid changes in carbon and phosphorus after rewetting of dry soil. Biology and fertility of Soils, 47(1), 41-50.
7. Chen, H., Jarosch, K. A., Mészáros, É., Frossard, E., Zhao, X., & Oberson, A. (2021). Repeated drying and rewetting differently affect abiotic and biotic soil phosphorus (P) dynamics in a sandy soil: A 33P soil incubation study. Soil Biology and Biochemistry, 153, 108079.
8. Chen, H., Lai, L., Zhao, X., Li, G., & Lin, Q. (2016). Soil microbial biomass carbon and phosphorus as affected by frequent drying-rewetting. Soil Research, 54(3), 321-327.

9. Cosentino, D., Chenu, C., & Le Bissonnais, Y. (2006). Aggregate stability and microbial community dynamics under drying–wetting cycles in a silt loam soil. Soil Biology and Biochemistry, 38(8), 2053-2062.
10. Ding, Z. J., Xu, Z., Tian, Y. B., Liu, K. W., Zhang, D. Y., Zhu, J. Q., & Hou, J. (2021). Reducing gas emissions from ratooning rice field using controlled nitrogen fertilization and alternate wetting-drying irrigation. Journal of Irrigation and Drainage, 40(7), 51-58. (in Chinese with English abstract)
11. Elmajdoub, B., & Marschner, P. (2021). Response of soil respiration and microbial biomass to drying and rewetting is greater in planted than in unplanted soil. Journal of Soil Science and Plant Nutrition, 21(4), 2765-2769.
12. Erinle, K. O., Bengtson, P., & Marschner, P. (2021). Rewetting intensity influences soil respiration and nitrogen availability. Journal of Soil Science and Plant Nutrition, 21(3), 2137-2144.
13. Erinle, K. O., Li, J., Doolette, A., & Marschner, P. (2018). Soil phosphorus pools in the detritusphere of plant residues with different C/P ratio—influence of drying and rewetting. Biology and Fertility of Soils, 54(7), 841-852.
14. Evans, S. E., & Wallenstein, M. D. (2012). Soil microbial community response to drying and rewetting stress: does historical precipitation regime matter? Biogeochemistry, 109(1), 101-116.
15. Fierer, N., & Schimel, J. P. (2002). Effects of drying-rewetting frequency on soil carbon and nitrogen transformations. Soil Biology and Biochemistry, 34(6), 777-787.
16. Gao, J., Feng, J., Zhang, X., Yu, F. H., Xu, X., & Kuzyakov, Y. (2016). Drying-rewetting cycles alter carbon and nitrogen mineralization in litter-amended alpine wetland soil. Catena, 145, 285-290.
17. Harrison-Kirk, T., Beare, M. H., Meenken, E. D., & Condron, L. M. (2013). Soil organic matter and texture affect responses to dry/wet cycles: Effects on carbon dioxide and nitrous oxide emissions. Soil Biology and Biochemistry, 57, 43-55.
18. Hu, Z., Chen, C., Chen, X., Yao, J., Jiang, L., & Liu, M. (2021). Home-field advantage in soil respiration and its resilience to drying and rewetting cycles.

Science of The Total Environment, 750, 141736.

19. Kruse, J. S., Kissel, D. E., & Cabrera, M. L. (2004). Effects of drying and rewetting on carbon and nitrogen mineralization in soils and incorporated residues. Nutrient Cycling in Agroecosystems, 69(3), 247-256.
20. Li, J. T., Wang, J. J., Zeng, D. H., Zhao, S. Y., Huang, W. L., Sun, X. K., & Hu, Y. L. (2018). The influence of drought intensity on soil respiration during and after multiple drying-rewetting cycles. Soil Biology and Biochemistry, 127, 82-89.
21. Li, J., Qu, W., Han, G., Lu, F., Zhou, Y., Song, W., ... & Eller, F. (2020). Effects of drying-rewetting frequency on vertical and lateral loss of soil organic carbon in a tidal salt marsh. Wetlands, 40(5), 1433-1443.
22. Liu, J., Xu, L., Yue, H., Ge, Z., Xu, W., Huang, X., Hu, Y. (2018). The characteristic of soil greenhouse gases emission at different vegetation restoration stages and its resposnes to wetting and drying alternation. Acta Agriculturae Universitatis Jiangxiensis, 40(6), 1331-1339. (in Chinese with English abstract)
23. Ma, G. Responses of CO_2 and N_2O emissions to alternating wetting and drying in alpine meadow ecosystem. Master Dissertation, Gansu Agricultural University, 2014. (in Chinese with English abstract)
24. Mavi, M. S., Marschner, P. (2012). Drying and wetting in saline and saline-sodic soils—effects on microbial activity, biomass and dissolved organic carbon. Plant soil, 355(1), 51-62.
25. Najera, F., Dippold, M. A., Boy, J., Seguel, O., Koester, M., Stock, S., ... & Matus, F. (2020). Effects of drying/rewetting on soil aggregate dynamics and implications for organic matter turnover. Biology and Fertility of Soils, 56(7), 893-905.
26. Ouyang, Y. & Li, X. Impacts of drying-wetting cycles on CO_2 and N_2O emissions from soils in different ecosystems. Acta Ecologica Sinica, 2013, 33(4), 1251-1259. (in Chinese with English abstract)
27. Ouyang, Y., & Li, X. (2020). Effect of repeated drying-rewetting cycles on soil extracellular enzyme activities and microbial community composition in arid and semi-arid ecosystems. European Journal of Soil Biology, 98, 103187.
28. Pezzolla, D., Cardenas, L. M., Mian, I. A., Carswell, A., Donovan, N., Dhanoa, M.

S., & Blackwell, M. S. (2019). Responses of carbon, nitrogen and phosphorus to two consecutive drying-rewetting cycles in soils. Journal of Plant Nutrition and Soil Science, 182(2), 217-228.

29. Rahman, M. T., Guo, Z. C., Zhang, Z. B., Zhou, H., & Peng, X. H. (2018). Wetting and drying cycles improving aggregation and associated C stabilization differently after straw or biochar incorporated into a Vertisol. Soil and Tillage Research, 175, 28-36.
30. Seneviratne, M., & Marschner, P. (2020). Soil respiration and nutrient availability after heating are influenced by salinity but not by prior drying and rewetting. Biology and Fertility of Soils, 56(5), 663-673.
31. Shah, A., & Gaebler, R. (2016). N_2O and CO_2 emissions from arable and grassland soils under various moisture regimes: a microcosm study. Malaysian Journal of Soil Science, 20, 95-110.
32. Shi, A., & Marschner, P. (2014). Drying and rewetting frequency influences cumulative respiration and its distribution over time in two soils with contrasting management. Soil Biology and Biochemistry, 72, 172-179.
33. Shi, A., & Marschner, P. (2015). The number of moist days determines respiration in drying and rewetting cycles. Biology and Fertility of Soils, 51(1), 33-41.
34. Shi, A., Marschner, P. (2014). Addition of a clay subsoil to a sandy topsoil changes the response of microbial activity to drying and rewetting after residue addition a model experiment. Journal of plant nutrient and soil science, 177(4), 532-540.
35. Shi, A., Yan, N., Marschner, P. (2015). Cumulative respiration in two drying and rewetting cycles depends on the number and distribution of moist days. Geoderma, 243, 168-174.
36. Sun, Q., Meyer, W. S., Koerber, G. R., & Marschner, P. (2015). Response of respiration and nutrient availability to drying and rewetting in soil from a semi-arid woodland depends on vegetation patch and a recent wildfire. Biogeosciences, 12(16), 5093-5101.
37. Wang, Y., Song, X. S., Wang, J., Yan, D. H., & Zhou, B. (2014). Effect of drying-rewetting alternation on soil carbon pool and mineralization of soil organic carbon.

Acta Pedologica Sinica, 51(2), 342-350. (in Chinese with English abstract)

38. Wu, J., & Brookes, P. C. (2005). The proportional mineralisation of microbial biomass and organic matter caused by air-drying and rewetting of a grassland soil. Soil Biology and Biochemistry, 37(3), 507-515.
39. Xiang, S. R., Doyle, A., Holden, P. A., & Schimel, J. P. (2008). Drying and rewetting effects on C and N mineralization and microbial activity in surface and subsurface California grassland soils. Soil Biology and Biochemistry, 40(9), 2281-2289.
40. Yang, F., Lee, X., Theng, B. K., Wang, B., Cheng, J., & Wang, Q. (2017). Effect of biochar addition on short-term N_2O and CO_2 emissions during repeated drying and wetting of an anthropogenic alluvial soil. Environmental Geochemistry and Health, 39(3), 635-647.
41. Yu, Z., Wang, G., & Marschner, P. (2014). Drying and rewetting–effect of frequency of cycles and length of moist period on soil respiration and microbial biomass. European Journal of Soil Biology, 62, 132-137.
42. Zeng, X., & Gao, Y. (2016). Short-term effects of drying and rewetting on CO_2 and CH4 emissions from high-altitude peatlands on the Tibetan Plateau. Atmosphere, 7(11), 148.
43. Zhang, C., Gao, Y., Wang, G., & Li, S. (2018). Effects of drying-wetting and additional nitrogen on CO_2 and N_2O emissions from farmland soils. Journal of Agro-Environment Science, 37(9), 2079-2090. (in Chinese with English abstract)
44. Zhang, X. Effect of drying-rewetting on litter and soil organic matter decomposition in Zoige Wetland. Master Dessertation, Beijing Forestry University, 2014. (in Chinese with English abstract)
45. Zhang, Y., & Marschner, P. (2016). Nutrient availability, soil respiration and microbial biomass after the second residue addition are influenced by the C/N ratio of the first residue added, but not by drying and rewetting between residue amendments. European Journal of Soil Biology, 77, 68-76.
46. Zhang, Z., Wang, D., & Li, M. (2022). Soil respiration, aggregate stability and nutrient availability affected by drying duration and drying-rewetting frequency. Geoderma, 413, 115743.

47. Zhu, B., & Cheng, W. (2013). Impacts of drying-wetting cycles on rhizosphere respiration and soil organic matter decomposition. Soil Biology and Biochemistry, 63, 89-96.
